# The Effects of Flavonoids on Skeletal Muscle Mass, Muscle Function, and Physical Performance in Individuals with Sarcopenia: A Systematic Review of Randomized Controlled Trials

**DOI:** 10.3390/nu15183897

**Published:** 2023-09-07

**Authors:** Cong Wu, Katsuhiko Suzuki

**Affiliations:** 1 Graduate School of Sport Sciences, Waseda University, Tokorozawa 359-1192, Japan; wucong86@foxmail.com; 2 Faculty of Sport Sciences, Waseda University, Tokorozawa 359-1192, Japan

**Keywords:** aging, nutrition, flavonoids, sarcopenia, skeletal muscle

## Abstract

Sarcopenia has become a significant obstacle to healthy aging in older adults. Flavonoids may contribute to treating sarcopenia, and attenuate the age-related loss of skeletal muscle mass, muscle strength, and physical function, however, their benefits in sarcopenic individuals remain unclear. This systematic review aimed to evaluate the effect of flavonoids on muscle mass, muscle strength, and physical performance in adults with sarcopenia based on randomized controlled trials (RCTs). This review was conducted in conformity with the Preferred Reporting Items for Systematic Reviews and Meta-Analyses (PRISMA) guidelines and the risk of bias was assessed using the Cochrane risk of bias tool. The article search was conducted using PubMed, Scopus, Embase, Cochrane, Web of Science databases, and Google Scholar for the period until June 2023. RCTs that assessed the effects of flavonoids/flavonoids combined with other supplementation/flavonoid-rich supplementations on skeletal muscle mass, muscle strength, and physical performance in adults diagnosed with sarcopenia before intervention were included. From the 309 articles found, a total of 6 RCTs met the inclusion criteria. RCTs evaluated the main outcomes of tea catechins, epicatechin, and isoflavones intervention. Skeletal muscle mass significantly increased in three studies, muscle strength significantly elevated in two studies, and physical performance significantly improved in two studies. The majority of studies (five in six) found at least one of the main outcomes is elevated by flavonoids intervention. Flavonoids may have a great potential to treat sarcopenia.

## 1. Introduction

Sarcopenia is characterized by a progressive decline in skeletal muscle mass and physical function, namely muscular strength and physical performance, that is commonly observed in older individuals. Multiple prospective studies have documented a decline in skeletal muscle mass of approximately 6% every decade following middle age [1]. This age-related condition has been shown to be on the rise in recent years [2]. Sarcopenia may lead to a variety of consequences, such as disability and increased mortality in the elderly, which has aroused widespread concern in society [3]. The risk of falling is increased by two–three times in the elderly when muscular strength declines as a result of muscle mass loss. Additionally, senior individuals with sarcopenia have a fall risk that is roughly 2.6 times greater than that of healthy elderly individuals, whereas elderly individuals with low muscle mass and muscular strength have mortality rates that are, respectively, 1.4 times and 2.34 times higher [4]. Consequently, the decline in skeletal muscle mass has resulted in major problems in the daily lives and overall well-being of older individuals [5]. The pathophysiology of sarcopenia is multifactorial. The process of aging disrupts the balance of skeletal muscle homeostasis, leading to an uneven distribution of anabolic and catabolic activities on the protein production pathway [6]. The development and progression of sarcopenia have been associated with many underlying risk factors. Physical fitness, inflammatory responses, antioxidants, mitochondrial function, hormones, the biochemistry of muscles, metabolic and cardiovascular disorders, neuromuscular junction dysfunction, insulin resistance, decreased number of satellite cells, and other clinical and physiological aspects of sarcopenia are discussed [7,8]. A previous study suggested that growth differentiation factor 8, p16 cyclin-dependent kinase 2A inhibitor, myogenic regulatory factors 4, and the stimulation of the Wnt pathway may lead to early sarcopenia development by changing satellite cell function, which would impair the restoring ability of skeletal muscle [9].

Presently, the Food and Drug Administration (FDA) has not approved any specific drugs to treat sarcopenia. Although several agents, such as appetite stimulants, growth hormone, myostatin inhibitors, activating II receptor drugs, β-receptor blockers, angiotensin-converting enzyme inhibitors, and troponin activators, are still recommended, the variable efficacy and side effects of these agents remain intractable [6]. 

Suitable protein intake and exercise are considered practical strategies to delay the progression of sarcopenia, given that there are no specific treatment options available for sarcopenia. However, a large number of patients suffering from sarcopenia are also combined with excess weight and osteoarthritis, which prevents them from maintaining regular and efficient levels of exercise. Due to their antioxidant, anti-inflammation, and anti-aging qualities with minimal toxicity, nutraceuticals and herbal medications have received a lot of interest recently for the prevention and treatment of sarcopenia [10].

Flavonoids are plant phenolic compounds with different medicinal properties such as antioxidant, anti-inflammation, and anti-cancer [11]. They are widely distributed in virtually every kind of plant, especially fruits and vegetables [12]. Also, flavonoids, which act as polyphenol antioxidants, are the main active components in many Chinese herbal medicines such as Alismatis rhizome and Achyranthis bidentate radix [13]. Flavonoids possess a fundamental chemical framework, including a 15-carbon skeleton, characterized by the presence of two phenyl rings (A and B) and a heterocyclic ring (C) that encompasses the oxygen atom [14]. Attributed to the C2=C3 functional double bond of the C ring in their chemical structure, flavonoids can be oxidized at different sites, leading to hydroxylation, methylation, and glycosylation [11]. Their variability functional group, degree of polymerization, degree of conjugation, and degree of substitution explain the broad and varied biological actions of flavonoids [11]. Depending on the chemical structure, flavonoids are divided into flavonols, flavan-3-ols, anthocyanidins, isoflavones, flavanones, and flavones [15]. Current evidence shows various flavonoids, such as rutin, nobiletin, luteolin, and quercetin have a favorable impact on skeletal muscle health by regulating protein homeostasis in vitro and in mouse models [16,17,18,19]. 

Although there was plenty of evidence showing the positive effect of flavonoids on muscle metabolism in animal. Not all clinical studies, nevertheless, have consistently shown a favorable effect [20,21,22,23]. Taking into account the aforementioned points, it should be indicated that an objective assessment of the effectiveness of flavonoids in the treatment of individuals with sarcopenia is needed. Therefore, we carried out a systematic review of randomized controlled trials (RCTs) to assess the effects of flavonoids on skeletal muscle mass, muscle function, and physical performance in sarcopenic adults. 

## 2. Materials and Methods

Following the Cochrane approach, we conducted a systematic search of papers and published our findings in conformity with Preferred Reporting Items for Systematic Reviews and Meta-Analyses (PRISMA) guidelines [24].

### 2.1. Search Strategy

In order to accomplish our objectives, the methodological approach for the current study was carried out utilizing the databases PubMed, Scopus, Embase, Cochrane, and Web of Science, as well as Google Scholar from inception up to June 2023. We performed the complete search in duplicate. We evaluated the eligibility of the title and abstract using predetermined inclusion and exclusion criteria. The full-text papers were checked for final inclusion if they were eligible. The following medical subject headings (MeSH) keywords were used: “Sarcopenia” AND (“2-Phenyl-Chromenes” OR “2 Phenyl Chromenes” OR “2-Phenyl-Benzopyran” OR “2 Phenyl Benzopyran” OR “2-Phenyl-Benzopyrans” OR “2 Phenyl Benzopyrans” OR “2-Phenyl-Chromene” OR “2 Phenyl Chromene” OR “Flavonoid” OR “Bioflavonoids” OR “Bioflavonoid” OR “Flavonoids” OR “Anthocyanins” OR “Benzoflavones” OR “beta-Naphthoflavone” OR “Biflavonoids” OR “Catechin” OR “Chalcones” OR “Flavanones” OR “Hesperidin” OR “Flavones” OR “Apigenin” OR “Diosmin” OR “Flavoxate” OR “Luteolin” OR “Flavonolignans” OR “Silymarin” OR “Silybin” OR “Flavonols” OR “Kaempferols” OR “Quercetin” OR “Rutin” OR “Hydroxyethylrutoside” OR “Isoflavones” OR “Coumestrol” OR “Genistein” OR ”Pterocarpans” OR “Rotenone” OR “Phloretin” OR “Polyphloretin Phosphate” OR “Proanthocyanidins”). The strategy of electronic searching is presented in detail in Appendix A.

### 2.2. Study Selection

Studies included in this systematic review were RCTs that assessed the effects of flavonoids, flavonoids combined with other supplementation, or flavonoid-rich supplementations (compared with placebo or control or other intervention) on sarcopenia. Studies assessed the effects of flavonoids (used alone or in combination) and flavonoid-rich supplementations in sarcopenic adults were included. We assessed potential publications for inclusion after evaluating titles, abstracts, and methodologies. If studies were duplicate records, reviews, other article types (letters, perspectives, commentaries, editorials, case reports, cohort studies, and cross-sectional studies), or full-text inaccessible, they were eliminated. Studies were excluded if they were conducted in non-sarcopenia patients, in silico, in vitro, animal studies, used administration other than flavonoids or were written in other languages than English. The search yielded a total of 309 publications: Web of Science (*n* = 51), PubMed (*n* = 44), Scopus (*n* = 87), Google Scholar (*n* = 71), Embase (*n* = 47), and Cochrane (*n* = 9). After the screening, 6 references were included.

Table 1 summarizes the patient, intervention/exposure, comparator, outcome, and study design (PICOS) criteria for studies to be included in and excluded from a systematic review.

### 2.3. Data Extraction

We evaluated the articles to determine if they should be included in the review and then extracted data into a standardized Microsoft Excel spreadsheet from the chosen articles. Relevant data were extracted, including year of publication, authorship, study location, participants’ gender, amount and condition, flavonoids type, type of study design, groups, dosage form, supplement dosage, intervention duration, outcome measurement, and main outcome.

### 2.4. Quality Assessment for Clinical Studies

With the following characteristics taken into account, we used the Cochrane Collaboration’s tool to evaluate the included RCTs for the risk of bias: (1) randomized sequence generation; (2) treatment allocation concealment; (3) participant blinding; (4) completeness of the outcome data; (5) selective outcome reporting; and (6) other sources of bias [25].

## 3. Results

**Figure 1:**
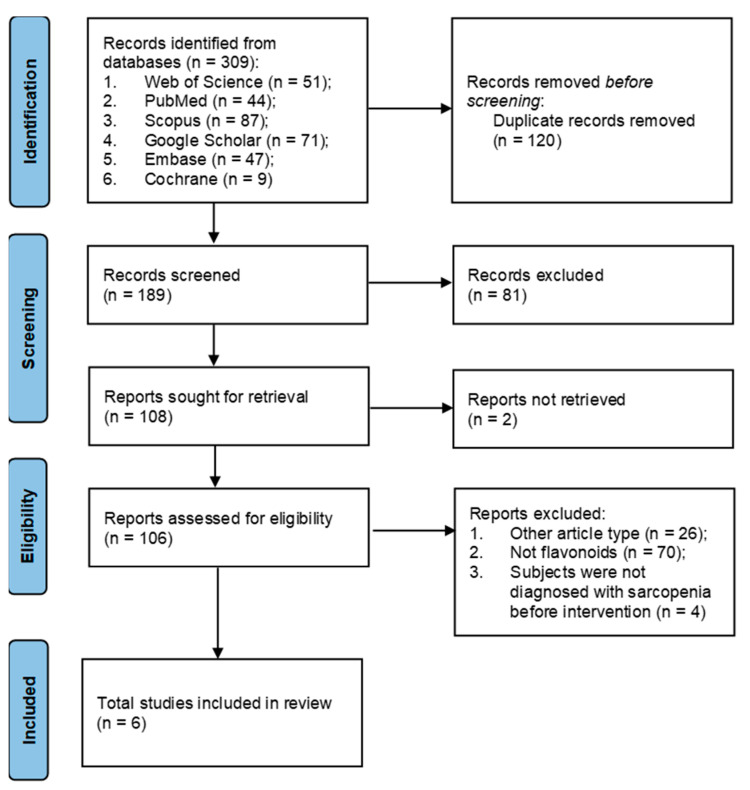
Flowchart of the selection process of the studies for systematic review.

There were 309 potentially eligible studies found after searching the Google Scholar, PubMed, Scopus, Embase, Cochrane, and Web of Science databases; 120 duplicate studies were eliminated. Based on title and methodology screening, and at least one of the following factors, a significant number of papers (183) were excluded: (1) reviews that were not filtered in the initial search; (2) in vitro, in silico, or in animal studies; (3) letters, commentaries, editorials, and case reports; (4) supplementation that does not contain any kind of flavonoids; or (5) full-text is not available. Figure 1 shows the study identification and selection procedures.

### 3.1. Study Characteristics

The characteristics of the studies are summarized in Table 2.

The study included publications that were published between 2007 and 2022. All subjects included in the six clinical trials were defined in sarcopenic conditions before intervention. A total of 440 participants were involved in these six RCTs and 81.6% of the subjects were female (359 female and 81 male). Four of the six included investigations [26,27,29,30] carried out in Asia, while two [28,31] were in the Americas. Two of the included RCTs [29,30] were conducted in a larger population of more than 100 participants, while the rest [26,27,28,31] were conducted in populations of less than 100 participants. This review included three RCTs [26,29,30] that assessed the effects of tea catechins (TCCs), two RCTs [27,28] that assessed the effects of epicatechin (EC), and one RCT [31] that assessed the effects of isoflavones. The duration of the trials ranged from 8 weeks to 6 months.

These RCTs included subjects with different diagnostic criteria. One study [26] referred to the Asia working group for sarcopenia (AWGS). One study [28] used the definition of the European Working Group on Sarcopenia in Older People (EWGSOP). One study [27] complied with Roubenoff’s view of sarcopenia [32]. The other three studies [29,30,31] did not describe their diagnostic criteria in detail, only that they used specific skeletal muscle index (SMI) values. Table 2 shows the different sarcopenia diagnostic criteria used in all the included studies.

### 3.2. Study Outcomes

The main outcomes of the studies are summarized in Table 3. Some outcomes measured by the included studies were not assessed in the present review. Among the studies with outcomes of interest, all included studies measured skeletal muscle mass, and five of six studies [26,27,28,29,30] measured muscle strength and physical performance as the main parameters to evaluate sarcopenia status. 

Four of the six studies [27,28,29,31] calculated SMI by dividing the estimated muscle mass by the square of the height to assess skeletal muscle mass. The other two studies [26,30] used estimated muscle mass measured by bioelectrical impedance analysis or dual-energy X-ray absorptiometry. Three of the five studies [26,29,30] assessed muscle strength by the measurement of maximal hand-grip strength and knee extension strength. One study [28] only measured maximal handgrip strength, and another study [27] used the measurement of maximal strength in leg press and chest press. For physical performance assessment, the included studies used the usual walking speed test, maximal walking speed test, Timed Up and Go test, six-minute walk test, step test, and sit-up test.

Based on the results presented by the authors of the RCTs included in the systematic review, three studies [26,27,31] showed flavonoids intervention improving skeletal muscle mass, two studies [28,29] showed flavonoids intervention improving muscle strength, and two studies [27,28] showed flavonoids intervention improving physical performance. Five of the involved six studies [26,27,28,29,31] found at least one of the outcomes is positively affected by flavonoids; it may be indicated that these studies presented effective flavonoids intervention for the sarcopenic individuals. 

### 3.3. Quality Assessment

**Figure 2:**
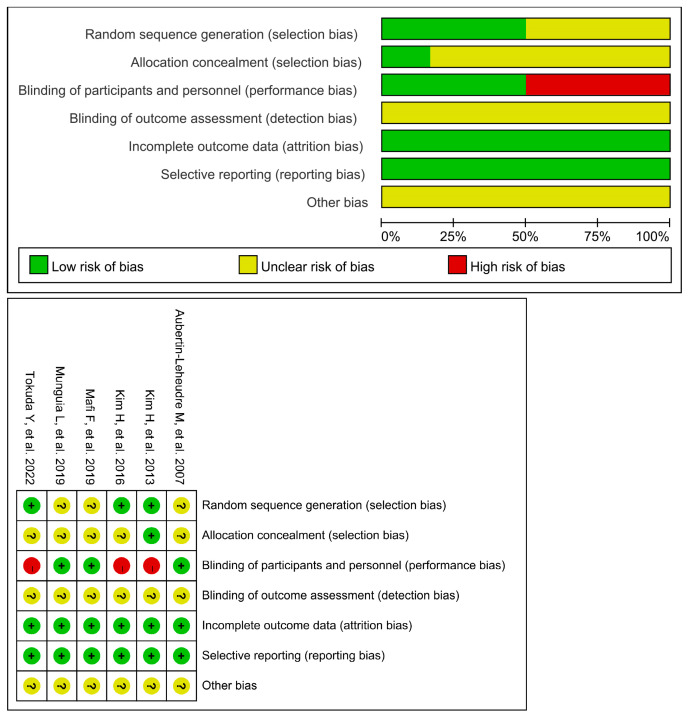
Risk of bias for all included studies [26,27,28,29,30,31].

Figure 2 displays the bias concerns associated with the studies. They usually had a low risk of bias for the majority of domains, such as completeness of the outcome, selective reporting, an unclear risk of bias for the blinding of outcome assessment, and other bias. In three of six studies [26,29,30], the random sequence generation was at low risk, while in other studies [27,28,31] it was unclear. In three studies [27,28,31], there was a low risk for participant and staff blinding, while there was a high risk in the rest of the three studies [26,29,30]. The allocation concealment was at a low risk in one study [30], and unclear in five studies [26,27,28,29,31]. Overall, three of the included studies [27,28,31] were assessed as ones with a medium risk of bias, and the other three studies [26,29,30] were indicated with a high risk of bias.

### 3.4. Flavonoids

Table 3 provides details on the impact of TCCs, EC, and isoflavones on skeletal muscle mass, muscle strength, and physical performance in sarcopenic adults.

## 4. Discussion

The purpose of this review was to assess the effect of flavonoids intervention on sarcopenic individuals. The included studies revealed that the majority of flavonoids interventions (five in six) turned out to be effective in improving skeletal muscle mass, muscle strength or physical performance in the group of sarcopenic individuals. In terms of risk of bias, three studies were indicated for a high risk of bias resulting from the participant blinding.

Numerous studies evaluated the effectiveness of flavonoids intervention in the prevention and management of sarcopenia among elderly adults not defined as sarcopenic before intervention. However, the outcomes are not as apparent as in the RCTs included in the present systematic review [22,23,33,34,35,36]. The interventions applied among sarcopenic individuals may be more effective than those applied in non-sarcopenic individuals. 

Catechins are the main secondary metabolites of tea, belonging to the group of flavonoids [37]. Catechins’ potential health benefits for cardiovascular disease and other chronic conditions have been widely studied [38]. The group of catechins (flavan-3-ol) include: (−)-epigallocatechin-3-gallate (EGCG), (−)-epicatechin-3-gallate, (−)-epigallocatechin, and EC [37]. These compounds exert anti-inflammatory effects by downregulating the expression of nuclear factor-κB and other proinflammatory cytokines [39]. According to the findings of Hung et al. [40], it was shown that EGCG exhibits stronger anti-inflammatory properties in comparison to other polyphenols found in green tea. Moreover, there were plenty of pre-clinical studies investigating the therapeutic effects of catechins on sarcopenia and the underlying mechanisms. Alway et al. [41] reported that catechins significantly attenuated the loss of hindlimb plantaris muscle mass, muscle fiber cross-sectional area, and tetanic force in the hindlimb suspension-induced sarcopenia rat model. The results demonstrated that catechins increased satellite cell proliferation and differentiation in skeletal muscles. Additionally, it was observed that catechins reduced oxidative stress and the abundance of the Bcl-2-associated X protein. Catechins have also been found to possess the ability to maintain muscle mass in aging rats. This effect is achieved, at least in part, by reducing protein degradation through the ubiquitin–proteasome pathway while simultaneously increasing the expression of anabolic factors [42]. 

In the present review, we include three studies [26,29,30] that used catechins (two studies [26,29] used catechins combined essential amino acid supplementation and one study [30] used catechins supplementation alone), and two studies [27,28] that used EC supplementation as an intervention, accounting for five out of six of the included studies. Catechins and the main compound appear to be the most promising flavonoids for treating sarcopenia. 

All five studies evaluated skeletal muscle mass, muscle strength, and physical performance. The studies by Tokuda et al. [26] showed a significantly greater increase in muscle mass in the exercise combined with supplementation (catechins + essential amino acids) group compared with the RE group. The studies by Kim et al. [29] showed that essential amino acids combined with catechins also improved muscle strength. The study of Kim et al. [30] evaluated the effects of catechins alone but did not show a significant effect on skeletal muscle mass, muscle strength, or physical performance unless combined with exercise. While these studies did not show anti-sarcopenia effects from catechins alone, they nonetheless revealed the anti-sarcopenia effects of a combination form of catechins and essential amino acids.

EC is the main compound of catechins. Mafi F et al. [27] and Munguia et al. [28] investigated how EC significantly elevated the SMI, muscle strength, and physical performance in sarcopenic individuals. Mafi F et al. showed that EC also significantly increased the follistatin/myostatin ratio, which improves muscle hypertrophic responses and may be related to the increase in skeletal muscle mass after EC intervention. Moreover, Munguia et al. found a significant decrease in malondialdehyde (lipid peroxidation marker), protein carbonylation, and interleukin-6 levels after flavonoids intervention. This indicated that flavonoids treatment alleviated oxidative stress and inflammatory endpoints, with positive effects on muscle strength and physical performance.

Isoflavones, which are plentiful in soybeans, are virtually solely generated by plants in the Leguminosae family, and the majority of them have estrogenic properties [43]. It is well known that soy isoflavones have a strong lipid-lowering effect, which improves vasodilation and regulates fasting glucose and insulin levels [44]. Phytoestrogens may also have advantageous effects on muscle mass because of their anti-inflammatory properties or estrogen receptor affinities [45]. Furthermore, the cohort study conducted by Wu et al. [46] revealed a significant correlation between increased regular intake of soy food and handgrip strength among the general Chinese adult population, suggesting that the consumption of isoflavone-rich soy foods may potentially contribute to the enhancement of muscle health. In the present review, the study conducted by Aubertin-Leheudre et al. [31] evaluated the effects of isoflavone containing daidzein, glycitein, and genistein in 18 sarcopenic women. Although this study showed isoflavone significantly increased skeletal muscle mass, the subjects included in this study were limited (*n* = 18) and uneven (12 in the isoflavone group and 6 in the placebo group). 

In this review, using consistent and specific criteria to define sarcopenia may be crucial in recruiting participants who had low levels of muscle mass and function to receive the expected benefit of flavonoids supplementation. However, only two of the included studies used AWGS and EWGSOP criteria. Although global diagnostic criteria for sarcopenia are now available, some included studies relied on expert scholarly views of sarcopenia at the time, which may have led to a risk of uncertain bias. Further RCTs that use specific operational criteria for sarcopenia are required. 

The present review provided an overview of the effectiveness of flavonoids interventions in the treatment of individuals diagnosed with sarcopenia based on the RCTs. There were several limitations of this study that must be indicated. Firstly, the small number of studies, sample size, and unbalanced gender ratio of the participants. Although a comprehensive search of six electronic databases has been undertaken, it is possible that some RCTs meeting the criteria of this review were overlooked, which may be present in another language or unpublished articles. Then, the discrepancy in participants’ recruitment and methodology could also have influenced the outcome of this study. Half of the included studies indicated a high risk of bias, which reduces the possibility of being able to draw conclusions. Only two studies included in this review conducted sarcopenia-related biochemical analyses to investigate the underlying mechanism of flavonoids intervention. Hence, the effect of flavonoids supplementation on skeletal muscle mass, strength, and physical performance in individuals with sarcopenia could be underestimated.

## 5. Conclusions

The majority of interventions studied within RCTs included in the systematic review, based on flavonoids/flavonoids combined with other supplementation/flavonoid-rich supplementations, were effective in the treatment of sarcopenia. 

In the future, more well-designed RCTs need to be conducted in this area to better identify the effectiveness and also reveal the underlying mechanism of the effect of flavonoids intervention in sarcopenic adults.

## Figures and Tables

**Table 1:** The criteria for a population, intervention/exposure, comparator, outcome, and study design (PICOS).

PICOS Parameter	Inclusion Criteria	Exclusion Criteria
Population	Subjects diagnosed with sarcopenia	Subjects not diagnosed with sarcopenia previously
Intervention/exposure	Intake of flavonoids/flavonoids combined with other supplementation/flavonoid-rich supplementations to treat sarcopenia	Used administration other than flavonoids
Comparison	Effectiveness of flavonoids, flavonoids combined with other supplementation, or flavonoid-rich supplementations vs. effectiveness of placebo/control/other intervention	Randomized controlled trials that combined both exercise and flavonoids intervention except where both control and intervention groups underwent the same exercise program
Outcome	Skeletal muscle mass, muscle strength, physical performance	Parameters for a mixed population of adults with and without sarcopenia
Study design	Randomized controlled trials	In silico, in vitro, animal studies, case reports, cohort studies, and cross-sectional studies

**Table 2:** Study details and participant characteristics of the six clinical trials.

Year	Author	Country	Subjects	Flavonoids Studied	Study Design	Sarcopenia Diagnose Criteria	Groups	Dosage Form	Dose	Intervention Duration
2022	Tokuda, Y. et al. [26]	Japan	46 older people (6 males, 40 females) aged 78~79 years with sarcopenia	TCCs	RCT	AWGS 2019 criteria: low hand-grip strength (males: <28.0 kg, females: <18.0 kg) or slow gait speed (males and females: <1.0 m/s), and low SMI (SMI, males: <7.0 kg/m^2^, females: <5.7 kg/m^2^).	(1) RE alone (RE) (2) RE followed by EAA ingestion (RE + EAA) (3) RE followed by EAA and TCCs supplementation (RE + EAA + TCC)	TCCs supplement powder	TCCs (540 mg/day, twice a week after RE)	24 weeks
2019	Mafi, F. et al. [27]	Iran	62 sarcopenic elderly males (mean age 68.63 ± 2.86 years) with class I sarcopenia	EC	RCT	Class I sarcopenia: AppMMI < 10.75 kg/m^2^	(1) RE Training (RT) (2) EC supplementation (EC) (3) RE Training + EC (RT + EC) (4) Placebo (PL)	EC capsules	EC (1 mg/kg body weight/day)	8 weeks
2019	Munguia, L. et al. [28]	Mexico	61 ndividuals (13 males and 48 females (mean age 75.9 ± 5.7 years) with sarcopenia	EC	RCT	SMI < 8.87 kg/m^2^ (men) and <6.42 kg/m^2^ (women)	(1) Highly alkalinized (no-flavonoid; NF) (2) Flavonoid-rich natural cocoa (F)	Flavonoid-rich natural cocoa beverage	179 mg flavonoids/day	8 weeks
2016	Kim, H. et al. [29]	Japan	137 female (mean age 80.9 ± 4.2~81.1 ± 5.1 years) defined with sarcopenia obesity	TCCs	RCT	Body fat percent > 32%, combined with SMI < 5.67 kg/m^2^ or grip strength < 17.0 kg or walking speed < 1.0 m/s	(1) Exercise only (Ex) (2) Exercise + EAA and TCCs supplementation (Ex + N) (3) EAA and TCCs supplementation (N) (4) Contorl (C)	Tea fortified with TCCs	One bottle of tea fortified with 540 mg of TCCs per day	3 months
2013	Kim, H. et al. [30]	Japan	116 women (mean age 79.6 ± 4.2~81.1 ± 3.7 years) were defined as sarcopenic	TCCs	RCT	At least one of the following condition: (1) AppMMI < 6.42 kg/m^2^ and knee extension strength < 1.01 Nm/kg; (2) AppMMI < 6.42 kg/m^2^ and walking speed < 1.10 m/s; (3) BMI < 22 and knee extension strength < 1.10 Nm/kg; (4) BMI < 22 and walking speed < 1.10 m/s	(1) Exercise and TCCs supplementation (Ex + TC) (2) Exercise (Ex) (3) TCCs supplementation (TC) (4) Contorl	Tea fortified with 540 mg of TCCs	One bottle of tea fortified with 540 mg of TCCs per day	3 months
2007	Aubertin-Leheudre, M. et al. [31]	Canada	18 sarcopenic–obese women (58 ± 5-year-old)	Isoflavones (daidzein, glycitein and genistein)	RCT	SMI < 6.87 kg/m^2^ and obesity as a body fat percent > 40%	(1) Isoflavone (ISO) (2) Placebo (PLA)	Capsules	70 mg i soflavones (44 mg of diadzein, 16 mg glycitein and 10 mg genestein)/day	24 weeks

Abbreviations: TCCs = tea catechins; RCT = randomized controlled trial; AWGS = Asia working group for sarcopenia; SMI = skeletal muscle index; RE = resistance exercise; EAA = essential amino acid; AppMMI = appendicular skeletal muscle mass index; EC = epicatechin; BMI = body mass index.

**Table 3:** Study main outcomes of the six clinical trials.

Author	Flavonoids Studied	Groups	Outcome Measurement	Main Outcome
Tokuda, Y. et al. [26]	TCCs	(1) RE alone (RE) (2) RE followed by EAA ingestion (RE + EAA) (3) RE followed by EAA and TCCs supplementation (RE + EAA + TCC)	(1) SMM: SMM measured by BIA (2) MS: maximum isometric hand-grip strength and knee extension strength (3) PP: usual walking speed	%Δ SMM was significantly greater in the RE + EAA + TCC group vs. RE group.
Mafi, F. et al. [27]	EC	(1) RE Training (RT) (2) EC supplementation (EC) (3) RE Training+EC (RT + EC) (4) Placebo (PL)	(1) SMM: SMI measured by DXA (2) MS: maximal strength in leg press and chest press (3) PP: TUG	(1) AppMMI significantly increased in RT + EC, RT, and EC vs. PL. The increases were significantly greater in RT + EC than EC. (2) Maximal MS of chest press and leg press significantly increased in RT + EC and RT, not in EC or PL. (3) TUG time significantly reduced in RT + EC, RT, and EC vs PL, also a significant difference between EC and RT + EC.
Munguia, L. et al. [28]	EC	(1) Highly alkalinized (no-flavonoid; NF) (2) Flavonoid-rich natural cocoa (F)	(1) SMM: SMI messured by BIA (2) MS: maximal handgrip strength (3) PP: six-minute walk test, step test, sit-up test, TUG	Flavonoids treatment significantly improved physical performance and muscle strength (F vs. NF)
Kim, H. et al. [29]	TCCs	(1) Exercise only (Ex) (2) Exercise + EAA and TCCs supplementation (Ex + N) (3) EAA and TCCs supplementation (N)(4) Contorl (C)	(1) SMM: SMI messured by BIA (2) MS: maximal grip strength, knee extension strength (3) PP: usual walking speed	(1) Significant increases in knee extension strength were observed in the Ex + N, Ex, and N groups (2) Usual walking increased in the Ex + N, but not in Ex, N and C group
Kim, H. et al. [30]	TCCs	(1) Exercise and TCCs supplementation (Ex + TC) (2) Exercise (Ex) (3) TCCs supplementation (TC) (4) Contorl (C)	(1) SMM: LBM, total and segmental muscle mass measured by BIA (2) MS: grip strength, knee extension strength (3) PP: usual and maximum walking speed, TUG	Combination of exercise and tea catechin supplementation had a beneficial effect on PP measured by walking ability and SMM vs. control but not significant in TC group.
Aubertin-Leheudre, M. et al. [31]	Isoflavones (daidzein, glycitein and genistein)	(1) Isoflavone (ISO) (2) Placebo (PLA)	SMM: SMI measured by DXA	Isoflavone significantly increased appendicular and leg LBM, SMI but not the placebo group.

Abbreviations: TCCs = tea catechins; RE = resistance exercise; EAA = essential amino acid; SMM = skeletal muscle mass; BIA = bioelectrical impedance analysis; MS = muscle strength; PP = physical performance; EC = epicatechin; DXA = dual-energy X-ray absorptiometry; SMI = skeletal muscle index; TUG = Timed Up and Go; AppMMI = appendicular skeletal muscle mass index; LBM = lean body mass.

## Data Availability

Not applicable.

## Appendix A

**Table A1:** The Detail of the Strategy of the Electronic Searching.

Database	The Detail of the Strategy of the Electronic Searching
Pubmed	(“sarcopenia”[Title/Abstract]) AND ((“2-Phenyl-Chromenes”[Title/Abstract]) OR (“2 Phenyl Chromenes”[Title/Abstract]) OR (“2-Phenyl-Benzopyran”[Title/Abstract]) OR (“2 Phenyl Benzopyran”[Title/Abstract]) OR (“2-Phenyl-Benzopyrans”[Title/Abstract]) OR (“2 Phenyl Benzopyrans”[Title/Abstract]) OR (“2-Phenyl-Chromene”[Title/Abstract]) OR (“2 Phenyl Chromene”[Title/Abstract]) OR (“Flavonoid”[Title/Abstract]) OR (“Bioflavonoids”[Title/Abstract]) OR (“Bioflavonoid”[Title/Abstract]) OR (“Flavonoids”[Title/Abstract]) OR (“Anthocyanins”[Title/Abstract]) OR (“Benzoflavones”[Title/Abstract]) OR (“beta-Naphthoflavone”[Title/Abstract]) OR (“Biflavonoids”[Title/Abstract]) OR (“Catechin”[Title/Abstract]) OR (“Chalcones”[Title/Abstract]) OR (“Flavanones”[Title/Abstract]) OR (“Hesperidin”[Title/Abstract]) OR (“Flavones”[Title/Abstract]) OR (“Apigenin”[Title/Abstract]) OR (“Diosmin”[Title/Abstract]) OR (“Flavoxate”[Title/Abstract]) OR (“Luteolin”[Title/Abstract]) OR (“Flavonolignans”[Title/Abstract]) OR (“Silymarin”[Title/Abstract]) OR (“Flavonols”[Title/Abstract]) OR (“Kaempferols”[Title/Abstract]) OR (“Quercetin”[Title/Abstract]) OR (“Rutin”[Title/Abstract]) OR (“Isoflavones”[Title/Abstract]) OR (“Coumestrol”[Title/Abstract]) OR (“Genistein”[Title/Abstract]) OR (“Pterocarpans”[Title/Abstract]) OR (“Rotenone”[Title/Abstract]) OR (“Phloretin”[Title/Abstract]) OR (“Polyphloretin Phosphate”[Title/Abstract]) OR (“Proanthocyanidins”[Title/Abstract]))
Scopus	TITLE-ABS-KEY ( “sarcopenia” AND ( “2-Phenyl-Chromenes” OR “2 Phenyl Chromenes” OR “2-Phenyl-Benzopyran” OR “2 Phenyl Benzopyran” OR “2-Phenyl-Benzopyrans” OR “2 Phenyl Benzopyrans” OR “2-Phenyl-Chromene” OR “2 PhenylChromene” OR “Flavonoid” OR “Bioflavonoids” OR “Bioflavonoid” OR “Flavonoids” OR “Anthocyanins” OR “Benzoflavones” OR “beta-Naphthoflavone” OR “Biflavonoids” OR “Catechin” OR “Chalcones” OR “Flavanones” OR “Hesperidin” OR “Flavones” OR “Apigenin” OR “Diosmin” OR “Flavoxate” OR “Luteolin” OR “Flavonolignans” OR “Silymarin” OR “Flavonols” OR “Kaempferols” OR “Quercetin” OR “Rutin” OR “Isoflavones” OR “Coumestrol” OR “Genistein” OR “Pterocarpans” OR “Rotenone” OR “Phloretin” OR “Polyphloretin Phosphate” OR “Proanthocyanidins” OR “Silybin” OR “Hydroxyethylrutoside”))
WOS	TS=(sarcopenic AND (2-Phenyl-Chromenes OR 2 Phenyl Chromenes OR 2-Phenyl-Benzopyran OR 2 Phenyl Benzopyran OR 2-Phenyl-Benzopyrans OR 2 Phenyl Benzopyrans OR 2-Phenyl-Chromene OR 2 Phenyl Chromene OR Flavonoid OR Bioflavonoids OR Bioflavonoid OR Flavonoids OR Anthocyanins OR Benzoflavones OR beta-Naphthoflavone OR Biflavonoids OR Catechin OR Chalcones OR Flavanones OR Hesperidin OR Flavones OR Apigenin OR Diosmin OR Flavoxate OR Luteolin OR Flavonolignans OR Silymarin OR Flavonols OR Kaempferols OR Quercetin OR Rutin OR Isoflavones OR Coumestrol OR Genistein OR Pterocarpans OR Rotenone OR Phloretin OR Polyphloretin Phosphate OR Proanthocyanidins OR Silybin OR Hydroxyethylrutoside))
Embase	sarcopenia:ti,ab,kw AND (‘2-phenyl-chromenes’:ti,ab,kw OR ‘2 phenyl chromenes’:ti,ab,kw OR ‘2-phenyl-benzopyran’:ti,ab,kw OR ‘2 phenyl benzopyran’:ti,ab,kw OR ‘2-phenyl-benzopyrans’:ti,ab,kw OR ‘2 phenyl benzopyrans’:ti,ab,kw OR ‘2-phenyl-chromene’:ti,ab,kw OR ‘2 phenyl chromene’:ti,ab,kw OR ‘flavonoid’:ti,ab,kw OR ‘bioflavonoids’:ti,ab,kw OR ‘bioflavonoid’:ti,ab,kw OR ‘flavonoids’:ti,ab,kw OR ‘anthocyanins’:ti,ab,kw OR ‘benzoflavones’:ti,ab,kw OR ‘beta-naphthoflavone’:ti,ab,kw OR ‘biflavonoids’:ti,ab,kw OR ‘catechin’:ti,ab,kw OR ‘chalcones’:ti,ab,kw OR ‘flavanones’:ti,ab,kw OR ‘hesperidin’:ti,ab,kw OR ‘flavones’:ti,ab,kw OR ‘apigenin’:ti,ab,kw OR ‘diosmin’:ti,ab,kw OR ‘flavoxate’:ti,ab,kw OR ‘luteolin’:ti,ab,kw OR ‘flavonolignans’:ti,ab,kw OR ‘silymarin’:ti,ab,kw OR ‘flavonols’:ti,ab,kw OR ‘kaempferols’:ti,ab,kw OR ‘quercetin’:ti,ab,kw OR ‘rutin’:ti,ab,kw OR ‘isoflavones’:ti,ab,kw OR ‘coumestrol’:ti,ab,kw OR ‘genistein’:ti,ab,kw OR ‘pterocarpans’:ti,ab,kw OR ‘rotenone’:ti,ab,kw OR ‘phloretin’:ti,ab,kw OR ‘polyphloretin phosphate’:ti,ab,kw OR ‘proanthocyanidins’:ti,ab,kw)
Cochrane	((“Sarcopenia”) AND (“Flavonoid” OR “Bioflavonoids” OR “Bioflavonoid” OR “Flavonoids” OR “Anthocyanins” OR “Benzoflavones” OR “beta-Naphthoflavone” OR “Biflavonoids” OR “Catechin” OR “Chalcones” OR “Flavanones” OR “Hesperidin” OR “Flavones” OR “Apigenin” OR “Diosmin” OR “Flavoxate” OR “Luteolin” OR “Flavonolignans” OR “Silymarin” OR “Silybin” OR “Flavonols” OR “Kaempferols” OR “Quercetin” OR “Rutin” OR “Hydroxyethylrutoside” OR “Isoflavones” OR “Coumestrol” OR “Genistein” OR “Pterocarpans” OR “Rotenone” OR “Phloretin” OR “Polyphloretin Phosphate” OR “Proanthocyanidins”)):ti,ab,kw
